# Sex prevalence of major congenital anomalies in the United Kingdom: A national population-based study and international comparison meta-analysis

**DOI:** 10.1002/bdra.23218

**Published:** 2014-02-12

**Authors:** Rachel Sokal, Laila J Tata, Kate M Fleming

**Affiliations:** Division of Epidemiology and Public Health, University of NottinghamNottingham, United Kingdom

**Keywords:** Congenital anomalies, Epidemiology, Prevalence, Risk factors, Sex

## Abstract

**Background:**

The aim of this study was to assess sex differences in major congenital anomaly (CA) diagnoses within a national population sample; to examine the influence of sociodemographic and maternal factors on these risks; and to conduct a meta-analysis using estimates from other population-based studies.

**Methods:**

We conducted a population-based study in a United Kingdom research database of prospectively collected primary care data (The Health Improvement Network) including children born 1990 to 2009 (*n* = 794,169) and identified major CA diagnoses using EUROCAT (European Surveillance of Congenital Anomalies) classification. Prevalence ratios (PR) were used to estimate the risk of CA in males compared with females for any CA, system-specific subgroups and specific CA diagnoses. In a subpopulation of children whose medical records were linked to their mothers', we assessed the effect of adjusting for sociodemographic and maternal factors on sex odds ratios. PRs were pooled with measures from previously published studies.

**Results:**

The prevalence of any CA was 307/10,000 in males (95% CI, 302–313) and 243/10,000 in females (95% CI, 238–248). Overall the risk of any CA was 26% greater in males (PR (male: female) 1.26, 95% CI, 1.23–1.30) however there was considerable variation across specific diagnoses. The magnitude and direction of risk did not change for any specific CA upon adjustment for sociodemographic and maternal factors. Our PRs were highly consistent with those from previous studies.

**Conclusion:**

The overall risk of CA is greater in males than females, although this masked substantial variation by specific diagnoses. Sociodemographic and maternal factors do not appear to affect these risks. Birth Defects Research (Part A) 100:79–91, 2014. © 2014 Wiley Periodicals, Inc.

## Introduction

Sex differences in several specific congenital anomalies (CA) have been documented as far back as the 1940s (Fogh-Andersen, 1942). Studies have reported conditions such as cleft lip and polydactyly to be more common in males (Hay, 1971), whereas neural tube defects (Deak et al., 2008) and cleft palate (Natsume et al., 2000) are reportedly more common in females. However, these initial observations were limited to one or a few specific diagnoses within small selected patient groups and the contributions of other risk factors with known associations with CAs and possible links to sex differentiation such as maternal age (Chahnazarian, 1988), smoking (Fukuda et al., 2002), diabetes (Rjasanowski et al., 1998), and epilepsy (James, 2000) were not examined.

Using large population-based resources such as birth or CA registries offers the potential to examine sex differences across the whole spectrum of CAs (Lary and Paulozzi, 2001). However, CA registries tend to be limited to specific geographical areas and vary in their methods of case ascertainment (Boyd et al., 2005) so may not all be representative of general populations. Additionally, they do not routinely collect comprehensive sociodemographic and maternal information nor details of children without CAs (Boyd et al., 2011) and, therefore, are unable to assess the potential impact of these factors on sex differences. Consequently, studies using these data may not be able to fully isolate sex-specific differences in CAs.

We aimed to estimate sex ratios in specific major CA diagnoses using a large source of routine healthcare data representative of the United Kingdom (U.K.) population and to examine the effects of sociodemographic and maternal factors on these ratios. We compared the direction and magnitude of our sex ratio estimates with those of published population-based studies obtained from a systematic search of the published literature and calculated pooled effect measures.

## Materials and Methods

### Study Population

The Health Improvement Network (THIN) is a computerized database of anonymized patient records from primary care in the United Kingdom. THIN data covers approximately 6% of the U.K. population (Cegedim Srategic Data, 2013). It is broadly representative of the U.K. population although is slightly more affluent (22.5% of THIN population live in most affluent areas compared with 20.0% of the national population according to national census-derived measures of household socioeconomic position) and includes a greater proportion of the population in the south of England (8.9%), Scotland (8.5%), and Wales (7.4%) compared with London (4.6%) and the Midlands (4.8%) (Blak et al., 2011). In September 2010, when the data used in this study were downloaded, 495 general practices were contributing data from over 9.5 million patients across the U.K. population. Our study population was all children in THIN born between 1990 and 2009 inclusive who were registered with their general practice before their first birthday.

### Case Definition and Classification of Congenital Anomalies

THIN collates information of patients' diagnoses and medical interventions at the time of consultation with primary care staff or from electronic and letter communication from secondary care institutions for diagnoses and tests that require complex assessment. All such records are entered into patients' electronic health records using the Read Clinical Classification system (Read codes), which is the standard coding system across all general practices in the United Kingdom. We identified all major CAs in children's general practice records using Read codes mapped to the European Surveillance of Congenital Anomalies (EUROCAT) classification system (EUROCAT, 2008) excluding CAs defined by EUROCAT as minor (EUROCAT, 2005). Congenital anomalies were categorized into 15 system-specific subgroups according to EUROCAT groupings (EUROCAT, 2008). These subgroups are broadly anatomical with an ‘Other' group, which includes disorders not included elsewhere such as skin disorders and situs inversus. We have published a full description of the case definitions and classification methodology elsewhere. In this work, we show across all system-specific groups (and specific CA diagnoses for the most prevalent system-specific subgroups) that prevalence estimates are comparable between THIN and the U.K. registries of the EUROCAT network and so demonstrate THIN to be a valid and complete source of data to investigate CAs in live-born children (Sokal et al., 2013).

### Statistical Analysis

Birth prevalence of CAs overall was calculated as the number of live-born children with one or more CA diagnoses per 10,000 live births for males and females separately and 95% confidence intervals (CIs) for these prevalence estimates were calculated based on a binominal distribution. We then calculated male and female prevalence estimates for each system-specific subgroup (one or more CA diagnosis within the same subgroup / 10,000 live births) and for specific CA diagnoses. We also calculated the prevalence of having a single, isolated CA diagnosis and the prevalence of multiple CA diagnoses (children with two or more CA diagnoses / 10,000 live births). Sex-specific chromosomal anomalies, e.g., Turner syndrome, and diagnoses of indeterminate sex, were excluded from analysis of specific CAs diagnoses but were included in the overall and system-specific subgroup prevalence measures. Prevalence ratios (PR) with 95% CIs were used to compare CA prevalence in males with females (M:F) at all levels described above.

To assess the potential effects of sociodemographic and maternal factors on these ratios, we used a subpopulation of children whose primary care medical records had been linked to those of their mothers' and where each mother had been registered with the practice before the estimated date of conception. Using logistic regression with a cluster term to adjust for multiple births to the same woman, we calculated the unadjusted odds ratios (OR) of any CA, system-specific CA subgroups and specific CA diagnoses in males compared with females and their 95% CIs. We then calculated adjusted ORs to account for the combined effect of factors available within our data that had established associations with CA and hypothesized links to sex differences, namely: socioeconomic status (SES, as measured by the Townsend Index of social and economic deprivation (Townsend et al., 1988), according to the child's home postcode), geographical area, year of birth, maternal age at delivery, maternal smoking status (at birth or in the year before pregnancy; classified as current smoker, ex-smoker, or never smoker); body mass index (BMI) before pregnancy (most recent BMI measure in the year before pregnancy), whether or not the mother had a diagnosis of diabetes before the pregnancy (defined as any code of type I or type II diabetes or insulin prescription), and whether or not the mother was prescribed any anti-epileptic drug in the 3 months before or in the first trimester of pregnancy. We compared the unadjusted and adjusted measures for any CA, each system-specific subgroup and specific CAs to identify changes of 5% or greater in the ORs of the two models.

All analyses were conducted using Stata v11 (StataCorp, Texas, U.S.A.).

### Systematic Review and Pooled Risk Measures

We systematically searched the literature using MEDLINE and EMBASE databases and references of selected papers to identify population-based studies that considered sex-differences in the prevalence of one or more specific CA using the following search terms: “sex” OR “sex differentiation” OR “sex difference*” AND “congenital anomal*” OR “congenital abnormal*” OR “birth defect*” OR “congenital malformation*”. We included original research published from 1960 until February 2013. We screened the titles, abstracts and full-text of papers to identify studies for inclusion in our review. We included papers which had assessed the sex prevalence of one or more CA at population level and excluded papers which had selected cases from patient populations. We compared the PRs and 95% CI for specific CAs across studies where they had been consistently defined in at least three studies including our own and calculated pooled measures of effect using published numerator and denominator data within the identified studies to calculate weighted averages.

In all analyses, we have reported 95% CIs as an indication of the precision of the estimate, however due to the number of outcomes in our study we have not interpreted them as an indication of statistical significance per se.

## Results

### Population Characteristics

In total, 794,169 children were included in the study population of whom 51.4% were male (Table 1). Of all children 21,931 had at least one diagnosis of a CA, a prevalence of 276 per 10,000 live births (95% CI, 273–280). There were 571,807 children in the subpopulation of children whose medical records were linked to those of their mother, representing 72% of our total population. This subpopulation was also 51.4% male. The distribution of children by year of birth, geographical area, SES, and maternal risk factors were similar in males and females (Table 1).

**Table 1:** Distribution of Sociodemographic Factors for the Total Population (*N* = 794,169) and of Maternal Factors for the Subpopulation^a^ (*N* = 571,807) for Male and Female Children Separately

	Male		Female			Male		Female	
	*n*	(%)	*n*	(%)		*n*	(%)	*n*	(%)
Total population	408,184	(51.4)	385,985	(48.6)	Subpopulation^*^	293,815	(51.4)	277,992	(48.6)
									
Any congenital anomaly	12,537	(3.1)	9,394	(2.4)	Any congenital anomaly	9,445	(3.2)	7,056	(2.5)
									
Sociodemographic factors					Maternal factors				
Geographical area					Age				
East Midlands	17,653	(4.3)	16,518	(4.3)	<20	17,808	(6.1)	16,775	(6.0)
East of England	33,182	(8.1)	31,145	(8.1)	20–24	52,316	(17.8)	49,349	(17.8)
London	43,453	(10.6)	42,087	(10.9)	25–29	86,503	(29.4)	81,729	(29.4)
North East	12,833	(3.1)	12,278	(3.2)	30–34	87,862	(29.9)	83,077	(29.9)
North West	40,832	(10.0)	38,537	(10.0)	35–39	41,549	(14.1)	39,643	(14.3)
Northern Ireland	15,929	(3.9)	15,292	(4.0)	>=40	7,777	(2.6)	7,419	(2.7)
Scotland	42,643	(10.4)	40,257	(10.4)					
South Central	48,841	(12.0)	45,433	(11.8)	Body mass index				
South East Coast	38,005	(9.3)	36,055	(9.3)	Normal	101,739	(34.6)	96,465	(34.7)
South West	39,576	(9.7)	37,550	(9.7)	Underweight	19,790	(6.7)	18,600	(6.7)
Wales	21,663	(5.3)	20,514	(5.3)	Overweight	36,765	(12.5)	34,953	(12.6)
West Midlands	36,752	(9.0)	34,500	(8.9)	Obese	17,512	(6.0)	16,698	(6.0)
Yorkshire & Humber	16,822	(4.1)	15,819	(4.1)	Morbidly obese	2,149	(0.7)	2,031	(0.7)
					Missing data	115,860	(39.4)	109,245	(39.3)
Year of birth									
1990–1994	85,614	(21.0)	79,842	(20.7)	Smoking status				
1995–1999	99,611	(24.4)	94,533	(24.5)	Non-smoker	66,524	(22.6)	62,924	(22.6)
2000–2004	104,623	(25.6)	99,476	(25.8)	Current smoker	36,942	(12.6)	35,167	(12.7)
2005–2009	118,336	(29.0)	112,134	(29.1)	Ex-smoker	88,804	(30.2)	84,116	(30.3)
					Missing data	101,545	(34.6)	95,785	(34.5)
SES (Townsend)									
1-most deprived	92,045	(22.5)	86,942	(22.5)	Diabetes				
2	76,761	(18.8)	72,300	(18.7)	Yes	1,693	(0.6)	1,498	(0.5)
3	78,583	(19.3)	74,373	(19.3)					
4	74,818	(18.3)	70,636	(18.3)	Epilepsy drugs				
5-least deprived	56,556	(13.9)	53,702	(13.9)	Yes	1,348	(0.5)	1,288	(0.5)
Missing	29,421	(7.2)	28,032	(7.3)					

a Subpopulation included only children whose medical records were linked to their mother's records and thus had available maternal factors for analysis (this was 72% of the total population). Distribution of sociodemographic factors were identical to the total population.

### Sex Prevalence

Numbers and prevalence of any CA and each system-specific subgroup for males and females in the total population are shown in Table 2 alongside the male to female (M:F) PRs. The prevalence of any CA was 307 per 10,000 in males (95% CI, 302–313) and 243 per 10,000 in females (95% CI, 238–248) with the overall risk of any CA being 26% greater in males than in females (PR [M:F]1.26; 95% CI, 1.23–1.30). We found the risk of multiple CAs in males compared with females to be even greater than that of a single diagnosis (one diagnosis PR [M:F] 1.19, 95% CI, 1.16–1.23; multiple diagnoses 1.53, 95% CI, 1.43–1.63).

**Table 2:** Number and Prevalence of System-Specific Congenital Anomalies by Sex and Male to Female (M:F) Prevalence Ratios (*N* = 794,169)

	Male			Female			Prevalence ratio	
	*N*	Prevalence (per 10,000 births)	95% CI	*N*	Prevalence (per 10,000 births)	95% CI	M:F	95% CI
Any congenital anomaly	12,537	307	(302, 313)	9,394	243	(238, 248)	1.26	(1.23, 1.30)
One isolated diagnosis	9,292	228	(223, 232)	7,378	191	(187, 196)	1.19	(1.16, 1.23)
Multiple diagnoses	2,192	53.7	(51.5, 56.0)	1,359	35.2	(33.4, 37.1)	1.53	(1.43, 1.63)
								
Any system specific								
Nervous System	657	16.1	(14.9, 17.4)	584	15.1	(13.9, 16.4)	1.06	(0.95, 1.19)
Eye	451	11.0	(10.1, 12.1)	376	9.7	(8.8, 10.8)	1.13	(0.99, 1.30)
Ear, face, and neck	105	2.6	(2.1, 3.1)	98	2.5	(2.1, 3.1)	1.01	(0.77, 1.33)
Heart	3,100	75.9	(73.3, 78.7)	3,060	79.3	(76.5, 82.1)	0.96	(0.91, 1.01)
Respiratory	286	7.0	(6.2, 7.9)	235	6.1	(5.3, 6.9)	1.15	(0.97, 1.37)
Orofacial clefts	647	15.9	(14.7, 17.1)	511	13.2	(12.1, 14.4)	1.20	(1.07, 1.34)
Digestive	453	11.1	(10.1, 12.2)	332	8.6	(7.7, 9.6)	1.29	(1.12, 1.49)
Abdominal wall	110	2.7	(2.2, 3.2)	108	2.8	(2.3, 3.4)	0.96	(0.74, 1.26)
Urinary	1,211	29.7	(28.0, 31.4)	750	19.4	(18.1, 20.9)	1.53	(1.39, 1.67)
Genital	2,879	70.5	(68.0, 73.2)	164	4.2	(3.6, 5.0)	16.6	(14.2, 19.4)
Limb	1,804	44.2	(42.2, 46.3)	2,408	62.4	(59.9, 64.9)	0.71	(0.67, 0.75)
Musculoskeletal	599	14.7	(13.5, 15.9)	416	10.8	(9.8, 11.9)	1.36	(1.20, 1.54)
Other malformations	395	9.7	(8.7, 10.7)	384	9.9	(9.0, 11.0)	0.97	(0.85, 1.12)
Chromosomal	764	18.7	(17.4, 20.1)	656	17.0	(15.7, 18.3)	1.10	(0.99, 1.22)
Genetic	354	8.7	(7.8, 9.6)	293	7.6	(6.7, 8.5)	1.14	(0.98, 1.33)

Prevalence ratios varied considerably across the 15 system-specific subgroups. The risk of CA was greater in males for five subgroups: genital (PR [M:F] 16.6; 95% CI, 14.2–19.4), urinary (1.53; 95% CI, 1.39–1.67), musculoskeletal (1.36; 95% CI, 1.20–1.54), and digestive (1.29; 95% CI, 1.12–1.49) disorders, and orofacial clefts (1.20; 95% CI, 1.07–1.34). The only subgroup which showed a lower risk in males was limb defects (PR [M:F] 0.71, 95% 0.67–0.75). There was little difference between sexes in the prevalence of CAs for the remaining system-specific subgroups.

The PR at a system-specific subgroup level masked considerable variation in prevalence ratios of specific diagnoses (Table 3). Nervous system anomalies showed little variation by sex except for hydrocephaly where the risk was 31% higher in males (PR [M:F] 1.31, 95% CI, 1.09–1.59). There were no apparent differences in the sex prevalence of specific diagnoses of eye or ear, face, and neck defects. For congenital heart anomalies, although we found little overall difference in risk between sexes at system level (PR [M:F] 0.96; 95% CI, 0.91–1.01), disorders categorized as “severe” according to EUROCAT classification appeared more prevalent in males than females (PR [M:F] 1.40; 95% CI, 1.23–1.58) driven by the higher risk in males of coarctation of aorta, transposition of great vessels and hypoplastic left heart. Aortic valve disorders were also more prevalent in males (PR [M:F] 2.12; 95% CI, 1.41–3.20); conversely atrial septal defects were more common in females (PR [M:F] 0.73; 95 CI, 0.65–0.82).

**Table 3:** Number and Prevalence of Specific Congenital Anomalies by Sex and Male to Female (M:F) Prevalence Ratios^a^

	Male			Female			Prevalence ratio	
	*N*	Prevalence (per 10,000 births)	95% CI	*N*	Prevalence (per 10,000 births)	95% CI	M:F	95% CI
Nervous system								
Hydrocephaly	253	6.2	(5.5, 7.0)	182	4.7	(4.1, 5.5)	1.31	(1.09, 1.59)
Microcephaly	191	4.7	(4.0, 5.4)	181	4.7	(4.0, 5.4)	1.00	(0.81, 1.22)
Neural tube defects	61	1.5	(1.1, 1.9)	75	1.9	(1.5, 2.4)	0.77	(0.55, 1.08)
Spina bifida	50	1.2	(0.9, 1.6)	57	1.5	(1.1, 1.9)	0.83	(0.57, 1.21)
Encephalocele	11	0.3	(0.1, 0.5)	16	0.4	(0.2, 0.7)	0.65	(0.30, 1.40)
Eye								
Congenital cataract	82	2.0	(1.6, 2.5)	79	2.0	(1.6, 2.6)	0.98	(0.72, 1.34)
Ano' / microphthalmos	67	1.6	(1.3, 2.1)	56	1.5	(1.1, 1.9)	1.13	(0.79, 1.61)
Anophthalmos	10	0.2	(0.1, 0.5)	5	0.1	(0.0, 0.3)	1.89	(0.65, 5.53)
Congenital glaucoma	33	0.8	(0.6, 1.1)	28	0.7	(0.5, 1.0)	1.11	(0.67, 1.84)
Ear, face, and neck								
Anotia	6	0.1	(0.1, 0.3)	<5	–	–	5.67	(0.68, 47.1)
Heart								
Ventricular septal defect	1,411	34.6	(32.8, 36.4)	1,379	35.7	(33.9, 37.7)	0.97	(0.90, 1.04)
Severe CHD	617	15.1	(13.9, 16.4)	418	10.8	(9.8, 11.9)	1.40	(1.23, 1.58)
Coarctation of aorta	159	3.9	(3.3, 4.6)	101	2.6	(2.1, 3.2)	1.49	(1.16, 1.91)
Tetralogy of Fallot	156	3.8	(3.2, 4.5)	131	3.4	(2.8, 4.0)	1.13	(0.89, 1.42)
Transposition of great vessels	102	2.5	(2.0, 3.0)	45	1.2	(0.9, 1.6)	2.14	(1.51, 3.04)
Atrioventricular septal defect	51	1.2	(0.9, 1.6)	56	1.5	(1.1, 1.9)	0.86	(0.59, 1.26)
Hypoplastic left heart	35	0.9	(0.6, 1.2)	12	0.3	(0.2, 0.5)	2.76	(1.43, 5.31)
Pulmonary valve atresia	29	0.7	(0.5, 1.0)	18	0.5	(0.3, 0.7)	1.52	(0.85, 2.74)
Total anom pulm venous return	23	0.6	(0.4, 0.8)	17	0.4	(0.3, 0.7)	1.28	(0.68, 2.39)
Ebstein anomaly	8	0.2	(0.1, 0.4)	15	0.4	(0.2, 0.6)	0.50	(0.21, 1.19)
Common arterial truncus	5	0.1	(0.0, 0.3)	<5	–	–	4.73	(0.55, 40.5)
Atrial septal defect	460	11.3	(10.3, 12.3)	596	15.4	(14.2, 16.7)	0.73	(0.65, 0.82)
Aortic valve atresia/stenosis	74	1.8	(1.4, 2.3)	33	0.9	(0.6, 1.2)	2.12	(1.41, 3.20)
Pulmonary valve stenosis	53	1.3	(1.0, 1.7)	51	1.3	(1.0, 1.7)	0.98	(0.67, 1.44)
Hypoplastic right heart	7	0.2	(0.1, 0.4)	<5	–	–	1.65	(0.48, 5.65)
Respiratory								
Choanal atresia	29	0.7	(0.5, 1.0)	30	0.8	(0.5, 1.1)	0.91	(0.55, 1.52)
Cystic adenomatous malf lung	5	0.1	(0.0, 0.3)	<5	–	–	1.18	(0.32, 4.40)
Cleft lip or palate								
Cleft lip with/out palate	384	9.4	(8.5, 10.4)	223	5.8	(5.0, 6.6)	1.63	(1.38, 1.92)
Cleft palate	263	6.4	(5.7, 7.3)	288	7.5	(6.6, 8.4)	0.86	(0.73, 1.02)
Digestive system								
Hirschsprung's disease	112	2.7	(2.3, 3.3)	45	1.2	(0.9, 1.6)	2.35	(1.67, 3.33)
Anorectal	105	2.6	(2.1, 3.1)	68	1.8	(1.4, 2.2)	1.46	(1.08, 1.98)
Esophageal atresia	62	1.5	(1.2, 1.9)	42	1.1	(0.8, 1.5)	1.40	(0.94, 2.07)
Oth. parts sm intestine	27	0.7	(0.4, 1.0)	32	0.8	(0.6, 1.2)	0.80	(0.48, 1.33)
Diaphragmatic hernia	15	0.4	(0.2, 0.6)	8	0.2	(0.1, 0.4)	1.77	(0.75, 4.18)
Duodenal	13	0.3	(0.2, 0.5)	26	0.7	(0.4, 1.0)	0.47	(0.24, 0.92)
Abdominal wall defects								
Gastroschisis	74	1.8	(1.4, 2.3)	85	2.2	(1.8, 2.7)	0.82	(0.60, 1.12)
Omphalocele	33	0.8	(0.6, 1.1)	23	0.6	(0.4, 0.9)	1.36	(0.80, 2.31)
Urinary system								
Hydronephrosis	154	3.8	(3.2, 4.4)	52	1.3	(1.0, 1.8)	2.80	(2.05, 3.83)
Renal dysplasia	78	1.9	(1.5, 2.4)	35	0.9	(0.6, 1.3)	2.11	(1.41, 3.14)
Bladder exstrophy/epispadias	77	1.9	(1.5, 2.4)	11	0.3	(0.1, 0.5)	6.62	(3.52, 12.5)
Posterior urethral valve	49	1.2	(0.9, 1.6)	<5	–	–	23.2	(5.63, 95.3)
Genital								
Hypospadias	2,581	63.2	(60.8, 65.7)	<5	–	–	814	(262, 2520)
Limb								
Club foot	632	15.5	(14.3, 16.7)	468	12.1	(11.1, 13.3)	1.28	(1.13, 1.44)
Hip dislocation/dysplasia	366	9.0	(8.1, 9.9)	1,318	34.1	(32.3, 36.0)	0.26	(0.23, 0.29)
Polydactyly	312	7.6	(6.8, 8.5)	211	5.5	(4.8, 6.3)	1.40	(1.17, 1.67)
Syndactyly	223	5.5	(4.8, 6.2)	136	3.5	(3.0, 4.2)	1.55	(1.25, 1.92)
Limb reduction	133	3.3	(2.7, 3.9)	129	3.3	(2.8, 4.0)	0.97	(0.77, 1.24)
Lower limb reduction	70	1.7	(1.3, 2.2)	89	2.3	(1.9, 2.8)	0.74	(0.54, 1.02)
Upper limb reduction	49	1.2	(0.9, 1.6)	29	0.8	(0.5, 1.1)	1.60	(1.01, 2.53)
Limb reduction other	18	0.4	(0.3, 0.7)	13	0.3	(0.2, 0.6)	1.31	(0.64, 2.67)
Arthrogryposis multi congenital	10	0.2	(0.1, 0.5)	9	0.2	(0.1, 0.4)	1.05	(0.43, 2.59)
Musculoskeletal								
Craniosynostosis	137	3.4	(2.8, 4.0)	35	0.9	(0.6, 1.3)	3.70	(2.55, 5.37)
Achondroplasia	37	0.9	(0.6, 1.2)	23	0.6	(0.4, 0.9)	1.52	(0.90, 2.56)
Other								
Disorders of skin	133	3.3	(2.7, 3.9)	89	2.3	(1.9, 2.8)	1.41	(1.08, 1.85)
Situs inversus	16	0.4	(0.2, 0.6)	14	0.4	(0.2, 0.6)	1.08	(0.53, 2.21)
Asplenia	9	0.2	(0.1, 0.4)	7	0.2	(0.1, 0.4)	1.22	(0.45, 3.26)
Chromosomal								
Down syndrome	407	10.0	(9.0, 11.0)	371	9.6	(8.7, 10.6)	1.04	(0.90, 1.19)
Wolf-Hirschhorn synd	9	0.2	(0.1, 0.4)	6	0.2	(0.1, 0.3)	1.42	(0.50, 3.99)
Edward syndrome	<5	–	–	10	0.3	(0.1, 0.5)	0.38	(0.12, 1.21)
Patau syndrome	<5	–	–	5	0.1	(0.0, 0.3)	0.76	(0.20, 2.82)

a Only CAs with at least ten cases are included in the table. Where the number of cases in a single sex is less than five, the PR has been displayed but the number and sex prevalence censored.

There were no apparent differences in the sex prevalence of specific respiratory anomalies. For cleft lip (with or without palate), we detected a risk over 60% higher in males compared with females (PR [M:F] 1.63; 95% CI, 1.38–1.92) but found a lower risk in males for cleft palate alone although CIs included the null value (PR [M:F] 0.86; 95% CI, 0.73–1.02). The PRs for digestive system disorders varied substantially across specific diagnoses. The risk of Hirschsprung's disease (PR [M:F] 2.35; 95% CI, 1.67–3.33) was greater in males compared with females whereas duodenal diagnoses were lower (PR [M:F] 0.47; 95% CI, 0.24–0.92). For abdominal disorders, the risk of gastroschisis was greater in females (PR [M:F] 0.82; 95% CI, 0.60–1.12) and the risk of omphalocele was greater in males (PR [M:F] 0.36; 95% CI, 0.80–2.31); however, the CIs for both these estimates were wide and included the null value. The risk of each specific urinary disorder was greater in males than in females.

Hypospadias accounted for 90% of genital disorders in males compared with just 2% in females. Excluding hypospadias from the overall risk measure for genital disorders reduced the PR (M:F) from 16.6 (95% CI, 14.2–19.4) to 1.83 (95% CI, 1.51–2.22). The overall lower risk of limb defects in males compared with females was driven by a substantial female preponderance of hip dislocation/dysplasia the risk of which was 74% lower in males (PR [M:F] 0.26; 95% CI, 0.23–0.29), whereas the risk of some other specific diagnoses was higher in males (e.g., club foot, polydactyly). For musculoskeletal disorders, the prevalence of craniosynostosis was nearly four times higher in males (PR [M:F] 3.70; 95% CI, 2.55–5.37) but other musculoskeletal diagnoses showed little variation by sex. Within the “Other” system-specific subgroup, skin disorders were more prevalent in males (PR [M:F] 1.41; 95% CI, 1.08–1.85). Within chromosomal disorders, the prevalence of Down syndrome did not differ between sexes (PR [M:F] 1.04; CI, 0.90–1.19).

### Adjustment for Sociodemographic and Maternal Factors

In the subpopulation of 571,807 children where we had access to children's medical records linked to those of their mothers', the birth prevalence of CA was 289 per 10,000 live births (95% CI, 284–293). The sex prevalence ratio of CAs for this subpopulation did not differ from the overall population (PR [M:F] 1.27; 95% CI, 1.23–1.31) compared with 1.26 (95% CI, 1.23–1.30) in the whole population of 794,169 children. Unadjusted odds of CA in males compared with females did not change by more than 2.5% upon adjustment for sociodemographic and maternal risk factors for any, each system-specific subgroup or specific CA (supplementary tables available online).

### Systematic Review and Pooled Risk Measures

**Figure 1:**
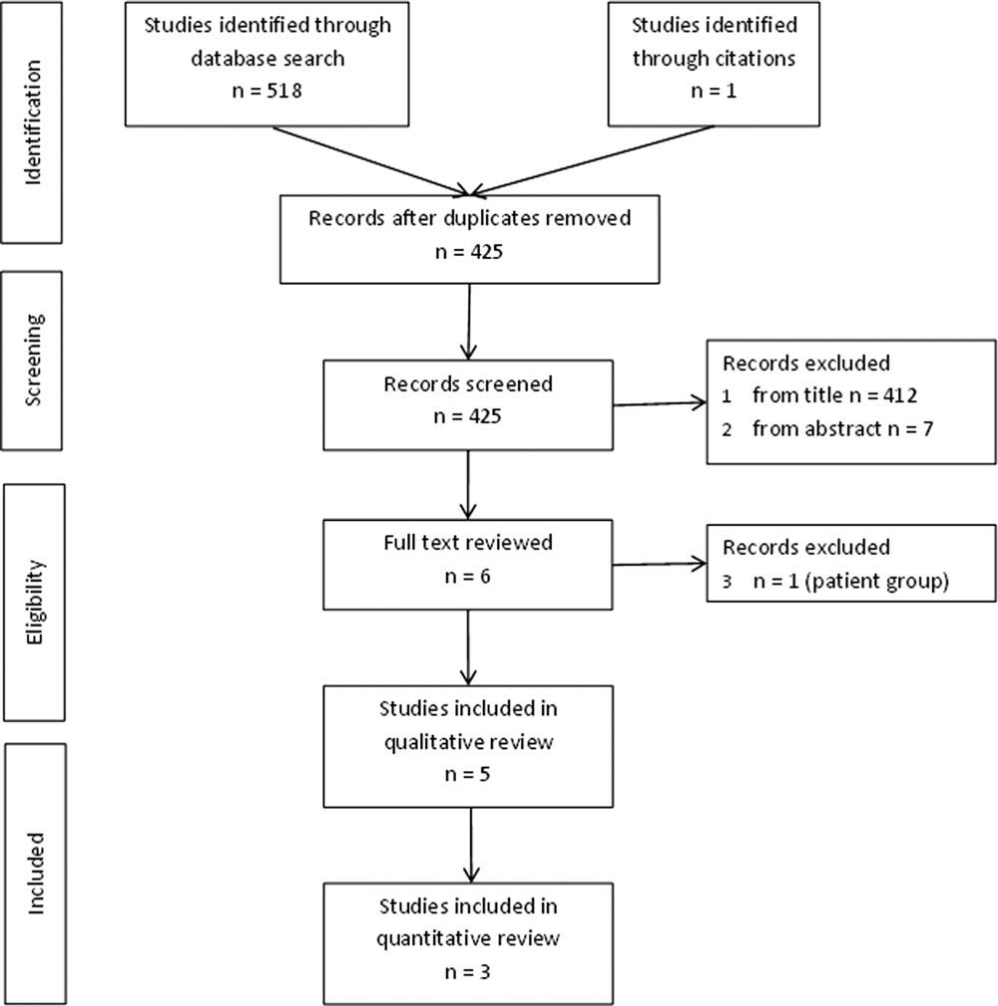
PRISMA flow diagram of the identification of studies included in the literature review.

We identified 425 publications in our search and reviewed the full text of six with five studies included in the final review (see Fig. 1) with the characteristics of these studies are presented in Table 4. The frequent use of some the search terms, e.g., “sex” and “congenital anomaly,” in the general key words of papers rather than as its specific focus resulted in a large number of papers returned in the search that were not relevant to the review, this led to the large volume of studies removed at the title stage. Nonrelevant papers typically did not assess sex differences in prevalence or had cases selected from patient populations. We chose the wide search term approach as we found using more specific search terms was too restrictive due to the variety of different terms used by authors. All included studies identified cases of congenital anomalies from either CA or birth registries: three studies used regional CA registries (Tennant et al. (2011), North of England, UK, 1998–2003; Shaw et al. (2003), Calfornia, USA 1989–1997; Lary & Paulozzi (2001), Atlanta, USA, 1968–1995); Lisi et al. (2005) used CA registry data from several international registries from 1968 to 1998 with registries contributing data at different times depending on the circumstances of each; and Rittler et al. (2004) identified cases from birth registry data from nine South American countries from 1982 to 1999. The size of the denominator population (size of population covered by registry) ranged from 646,174 (Tennant et al., 2011) to 2,537,001 (Shaw et al., 2003) and the total numerator (number of cases of CAs) size ranged from 12,795 (Tennant et al., 2011) to 55,422 (Shaw et al., 2003), although these figures were not available for all studies (see Table 4). Due to the potential of overlapping cases and baseline populations in two of the publications with those of our study and the absence of denominator data in one, we excluded two studies from the pooled analysis (Lisi et al., 2005; Tennant et al., 2011).

**Table 4:** Comparison of Sex Prevalence (PR, Male:Female) Ratios for Congenital Anomalies in Population-Based Studies and Pooled Estimates a

	Current study		Tennant et al. 2011		Lisi et al. 2005		Rittler et al. 2004		Shaw et al. 2003		Lary & Paulozzi 2001		Pooled^b^	
	PR	95% CI	PR	95% CI	PR^*^		PR	95% CI	PR	95% CI	PR	95% CI	PR	95% CI
Total baseline population (N)		794,169		646,174		NK		1,432,220		2,537,001		853,456		
Total children with CA (*n*)		21,931		12,795		NK		NK		55,422		28,965		
Any CA	1.26	(1.23, 1.30)	1.15	(1.11, 1.19)					1.42	(1.39, 1.44)	1.38	(1.35, 1.41)	1.37	(1.36, 1.39)
Multiple (two or more) diagnoses	1.52	(1.44, 1.61)	1.43	(1.23, 1.67)					0.69	(0.52, 0.91)				
Nervous system														
Hydrocephaly	1.31	(1.09, 1.59)	1.32	(0.97, 1.79)		1.23	1.34	(1.16, 1.54)	1.02	(0.92, 1.13)	1.27	(1.07, 1.51)	1.17	(1.09, 1.26)
Microcephaly	1.00	(0.81, 1.22)	0.73	(0.48, 1.10)			0.61	(0.46, 0.81)	0.59	(0.54, 0.64)	0.67	(0.56, 0.81)	0.63	(0.59, 0.68)
Spina bifida	0.83	(0.57, 1.21)	0.83	(0.69, 0.99)		0.86	0.72	(0.64, 0.81)	0.72	(0.61, 0.86)	0.77	(0.60, 0.99)	0.73	(0.67, 0.80)
Encephalocele	0.65	(0.30, 1.40)	0.75	(0.45, 1.25)		0.72			0.83	(0.64, 1.08)				
Arhinen'/holoprosencephaly	0.47	(0.09, 2.58)	0.6	(0.31, 1.18)		0.61								
Eye														
Congenital cataract	0.98	(0.72, 1.34)	0.95	(0.54, 1.67)					0.86	(0.70, 1.05)				
Ano'/microphthalmos	1.13	(0.79, 1.61)	1.7	(0.57, 5.08)					0.61	(0.53, 0.71)				
Anophthalmos	1.89	(0.65, 5.53)				1.08			0.73	(0.47, 1.15)				
Ear, face, and neck														
Anotia	5.67	(0.68, 47.1)	2.6	(0.83, 8.17)		1.58								
Heart	0.96	(0.91, 1.01)					1.01	(0.92, 1.10)			1.07	(1.02, 1.12)	1.02	(0.99, 1.05)
Ventricular septal defect	0.97	(0.90, 1.04)	0.95	(0.87, 1.04)			0.87	(0.74, 1.02)	0.89	(0.84, 0.95)			0.92	(0.88, 0.96)
Coarctation of aorta	1.49	(1.16, 1.91)	1.68	(1.25, 2.27)		1.31			1.29	(1.13, 1.47)				
Tetralogy of Fallot	1.13	(0.89, 1.42)	1.28	(0.96, 1.71)		1.2			1.08	(0.94, 1.25)				
Transposition of great vessels	2.14	(1.51, 3.04)	2.01	(1.49, 2.72)		1.91			1.61	(1.42, 1.81)	1.38	(1.14, 1.68)	1.58	(1.44, 1.75)
Atrioventricular septal defect	0.86	(0.59, 1.26)	0.74	(0.50, 1.10)					0.67	(0.59, 0.77)				
Hypoplastic left heart	2.76	(1.43, 5.31)	1.45	(0.99, 2.13)		1.60			1.23	(1.01, 1.48)	1.34	(1.02, 1.76)	1.32	(1.14, 1.54)
Ebstein anomaly	0.50	(0.21, 1.19)	1.08	(0.65, 1.80)					0.91	(0.67, 1.24)				
Common arterial truncus	4.73	(0.55, 40.5)	1.82	(0.93, 3.56)					0.99	(0.75, 1.30)				
Atrial septal defect	0.73	(0.65, 0.82)	0.61	(0.49, 0.75)					1.00	(0.95, 1.05)	0.68	(0.51, 0.91)	0.94	(0.90, 0.99)
Aortic valve atresia/stenosis	2.12	(1.41, 3.20)	2.50	(1.78, 3.49)					1.22	(1.03, 1.46)	1.58	(1.15, 2.17)	1.39	(1.20, 1.60)
Respiratory														
Choanal atresia	0.91	(0.55, 1.52)	0.71	(0.25, 2.05)					0.91	(0.73, 1.14)				
Cystic adenomatous malf lung	1.18	(0.32, 4.40)	0.77	(0.37, 1.60)					1.50	(0.87, 2.60)				
Cleft lip and palate														
Cleft lip with/out palate	1.63	(1.38, 1.92)	1.81	(1.47, 2.24)		1.68	1.29	(1.15, 1.44)	1.27	(1.17, 1.37)			1.32	(1.24, 1.40)
Cleft palate	0.86	(0.73, 1.02)	0.75	(0.58, 0.98)		0.73	0.57	(0.43, 0.75)	0.67	(0.60, 0.75)			0.71	(0.65, 0.77)
Digestive system														
Hirschsprung's disease	2.35	(1.67, 3.33)	2.9	(1.62, 5.20)					2.69	(2.11, 3.42)	2.79	(1.95, 3.99)	2.63	(2.21, 3.12)
Anorectal	1.46	(1.08, 1.98)	3.21	(1.90, 5.43)		1.78								
Esophageal atresia	1.40	(0.94, 2.07)	1.37	(0.93, 2.04)		1.30								
Oth. parts sm intestine	0.80	(0.48, 1.33)	1.70	(0.57, 5.08)		0.92								
Diaphragmatic hernia	1.77	(0.75, 4.18)	1.51	(1.11, 2.05)		1.34	1.12	(0.87, 1.45)			1.48	(1.11, 1.96)	1.29	(1.07, 1.55)
Abdominal wall defects														
Gastroschisis	0.82	(0.60, 1.12)	0.93	(0.71, 1.22)		0.97	0.90	(0.69, 1.17)						
Omphalocele	1.36	(0.80, 2.31)	0.97	(0.61, 1.55)		1.22								
Urinary system														
Hydronephrosis	2.80	(2.05, 3.83)	3.18	(2.49, 4.05)			2.92	(2.33, 3.66)			2.11	(1.74, 2.56)	2.50	(2.19, 2.85)
Bladder exstrophy/epispadias	6.62	(3.52, 12.5)	1.22	(0.45, 3.27)					0.48	(0.12, 1.91)				
Limb														
Club foot	1.28	(1.13, 1.44)					1.68	(1.51, 1.87)	0.91	(0.81, 1.04)	1.75	(1.46, 2.10)	1.35	(1.27, 1.44)
Hip dislocation/dysplasia	0.26	(0.23, 0.29)							0.31	(0.27, 0.37)	0.33	(0.28, 0.39)	0.29	(0.27, 0.32)
Polydactyly	1.40	(1.17, 1.67)	2.05	(1.03, 4.06)		1.30	1.24	(1.14, 1.34)	1.20	(1.10, 1.31)	1.45	(1.28, 1.63)	1.27	(1.21, 1.34)
Syndactyly	1.55	(1.25, 1.92)	2.08	(0.72, 5.99)			1.92	(1.54, 2.38)	1.43	(1.30, 1.57)	1.75	(1.42, 2.17)	1.53	(1.42, 1.65)
Lower limb reduction	0.74	(0.54, 1.02)	1.20	(0.69, 2.11)					1.19	(0.95, 1.50)				
Upper limb reduction	1.60	(1.01, 2.53)	1.07	(0.76, 1.52)					1.03	(0.89, 1.19)				
Musculoskeletal														
Craniosynostosis	3.70	(2.55, 5.37)	1.77	(0.94, 3.31)							2.04	(1.63, 2.54)		
Chromosomal														
Down syndrome	1.04	(0.90, 1.19)				1.05	1.00	(0.93, 1.09)			1.16	(1.01, 1.33)	1.04	(0.98, 1.11)
Edward syndrome	0.38	(0.12, 1.21)	0.7	(0.53, 0.91)		0.72			0.63	(0.49, 0.80)				
Patau syndrome	0.76	(0.20, 2.82)	0.95	(0.63, 1.43)		0.99			0.30	(0.21, 0.45)				

a Congenital anomalies included in table if sex prevalence ratio published in current and at least two other studies.

b Pooled measures calculated where data available in current and at least two other studies excluding Tennant *et al*. and Lisi *et al*. due to the potential of shared population in both and no denominator data in the study from Lisi *et al*.

Current study: general practice population; UK-wide; 1990–2009. Tennant et al.: CA registry; North of England, UK; 1998–2003.

Lisi et al.: CA registry; international (19 registries: Europe n=13, Americas n=3, other n=3); 1968–1998. (^*^No denominator data included; published sex ratios adjusted for the study's referent birth sex ratio of 1.06 thus 95% confidence intervals not known).

Rittler et al.: birth cohort; South America (9 countries); 1982–1999.

Shaw et al.: CA registry; California, USA; 1989–1997.

Lary & Paulozzi: CA registry; Atlanta, USA; 1968–1999.

NK – not known.

The PR (M:F) for any CA in our study was consistent with those of others. Lary and Paulozzi (2001) calculated a male to female prevalence ratio of 1.38 (95% CI, 1.35–1.41) and Shaw et al. (2003) calculated 1.42 (95% CI, 1.39–1.44); we calculated a pooled PR (M:F) of 1.37 (95% CI, 1.36–1.39) for any major CA. In the only published U.K.-based study, Tennant et al. (2011) reported a M:F prevalence ratio of 1.15 (95% CI, 1.11–1.19) for the North of England congenital anomaly register (NORCAS), however, this study omitted sex-linked urinary and genital anomalies in addition to chromosomal anomaly diagnoses from their analysis and when recalculated our overall PR with these exclusions it was 1.02 (95% CI, 0.99–1.05).

There was good agreement across studies for the majority of sex prevalence ratios of specific CAs with the precision of these estimates increasing in the pooled estimates. For example, the PR (M:F) for spina bifida PR ranged from 0.72–0.86 in individual studies with a pooled PR of 0.73 (95% CI, 0.67–0.80) and the PR (M:F) for cleft lip PR ranged from 1.27–1.81 in individual studies with a pooled PR 1.32 (95% CI, 1.24–1.40).

For many CAs PRs were consistent across the studies with each measure close to the null value. The pooled estimates indicated potential differences in the sex prevalence of several of these anomalies, for example ventricular septal defect PR (M:F) 0.92 (95% CI, 0.88–0.96), whereas for others the risk remained equivocal between the sexes, e.g., Down syndrome PR (M:F) 1.04 (95% CI, 0.98–1.11). Where there was greater variation in the point estimates between studies, this tended to be due to smaller numbers of cases in rarer conditions with large CIs which overlapped between studies, for example Ebstein anomaly and cystic adenomatous malformation of the lung. For many of these individual estimates, we were not able to calculate pooled estimates as individual PRs were not reported in at least three separate studies.

## Discussion

We found that the prevalence of congenital anomalies was 26% higher in males compared with females (PR [M:F] 1.26; 95% CI, 1.23–1.30); however, this masked variation across system-specific subgroups and specific diagnoses. Adjusting for some important sociodemographic and maternal risk factors known to be associated with prevalence of congenital anomalies and potentially to sex differentiation did not change the magnitude of the ORs for any specific CA or at a system level. Our results were highly consistent with those from previous studies (Lary and Paulozzi, 2001; Shaw et al., 2003; Rittler et al., 2004; Lisi et al., 2005; Tennant et al., 2011).

This is the first study of a national U.K.-based population to examine sex differences in congenital anomalies. The pooled measures we calculated increased the confidence in the estimates compared with any single study. Our study, alongside others, has shown a greater number of CAs in which there is a male predominance rather than a female one. For CAs that have a greater predominance in males, the sex-specific risks tend to be of larger magnitude (PR [M:F] >1.40), whereas for conditions in which there is a female dominance the risk differences between sexes tend to be smaller (PR [M:F] between 0.80 and 1.00). These smaller differences are, therefore, statistically more difficult to detect, but the consistency of female predominance across studies and the pooled measures we calculated support the likelihood of existing differences in the prevalence between the sexes. As these risk differences are statistically small, they may not be immediately important in clinical practice yet they may offer further clues to the causative mechanisms underlying these CAs as, despite the consistency of sex ratios across studies, there remains little clear evidence from physiological, biological, or endocrinological research to explain these differences. These differences may also contribute to potential explanations for some sex differences in later adult disease outcomes that may have been initially related to CAs.

Primary care data have been shown to be a valid and complete source of data in which to investigate congenital anomalies (Wurst et al., 2007a, 2007b; Charlton et al., 2010, 2011; Sokal et al., 2013). Using a U.K.-wide sample of primary care data avoids some of the biases in case ascertainment and data collection that can affect studies using registry data (Rankin et al., 2005), although as it is not possible to verify individual diagnoses within these data, it is possible that some misclassification or under-ascertainment exists. All of the other population-based studies we identified and included in this review have utilized birth cohort or CA registry data. This means that, although they often study large populations, they have either been limited to specific registries and therefore geographic areas (Lary and Paulozzi, 2001; Shaw et al., 2003; Rittler et al., 2004; Tennant et al., 2011) or conversely, have combined several international sources (Lisi et al., 2005). Although there are obvious limitations of analyses within single sources, the combination of multiple registries also can be problematic as in addition to demographic and environmental differences between populations, case ascertainment and diagnostic criteria may also differ between sources (Loane et al., 2011). Consequently, the methodological validity of pooling multiple sources in such a way to provide overall measures of sex differences may not be robust. Despite this possible limitation, we in fact found estimates to be remarkably similar across all the studies included in the review suggesting that this potential heterogeneity does not appear to affect sex differences in CAs. This may in turn give further clues to causative mechanisms by indicating factors such as demographic and healthcare differences between populations may not be important mediators in the sex differences seen in CA prevalence.

Although our study was not able to include CAs diagnosed in pregnancies ending in stillbirth, termination of pregnancy or spontaneous abortion, using information on pregnancies resulting in live births only is consistent with the majority of the population-based studies we identified (Lary and Paulozzi, 2001; Shaw et al., 2003; Rittler et al., 2004; Lisi et al., 2005). Only Tennant and colleagues (2011) were able to include such cases in their analysis, and the inclusion of these CAs in their analysis did not result in substantially different PRs to those of other studies. There is however some evidence to suggest that there may be selective late-miscarriage of males with neural tube defects (Källén et al., 1994) and sex-differences in survival of fetuses may exist for other CAs (Lubinsky, 1997), thus real differences in sex prevalence from conception may not be detected in studies limited to live births only. Notwithstanding this, it is not possible to know the number of pregnancies that spontaneously abort at an early stage of pregnancy due to congenital anomaly rendering it impossible to ever ascertain a measure of prevalence that is completely free from this bias.

This is the first U.K. study to control for the effect of potentially confounding variables. Our analysis indicated sociodemographic and maternal factors did not affect the sex prevalence of any CA, akin to the results found in the work by Shaw et al. (2003) from California, U.S., where adjustment for parity, maternal age, education, and ethnicity did not affect the sex prevalence of CAs. We acknowledge that the recording of some of these maternal risk factors in our data was not complete, for example, smoking status preconception, however we have been able to examine many more risk factors than previously seen in the only other study examining maternal risk factors. As the proportions of missing data were identical between boys and girls in our population, it is unlikely that this would have had any substantial impact on the findings. Although we do not have reason to believe that the sex ratio of those with missing data should differ from those without, we included missing data as a separate category in our adjusted models so the potential for residual confounding should be considered when comparing unadjusted ORs with those adjusted for sociodemographic and maternal factors. For specific level diagnoses, there is of course the issue of lack of power, thus we cannot conclude definitively that these maternal risk factors do not influence the sex prevalence ratio for specific CA diagnoses. Furthermore, although we were able to control for several important variables, we did not have data available to directly measure the effect of some other factors such as maternal stress, which may be associated with sex differentiation of fetuses (Chason et al., 2012) nor did we attempt to adjust for maternal factors where the evidence of an association with CAs in offspring remains equivocal, for example, selective serotonin reuptake inhibitors (Diav-Citrin and Ornoy, 2012).

Despite the consistency of findings across studies of CA sex prevalence ratios, there remains little evidence from physiological, biological, or endocrinological research to explain differences. Studies have found a low male to female sex ratio of spontaneously aborted fetuses with an anomaly compared with those without suggesting a better survival rate to birth of male fetuses with anomalies compared with females which could contribute to a higher risk in live born children (Hay, 1971; Kellokumpu-Lehtinen and Pelliniemi, 1984; Khoury et al., 1989). However, this pattern appears to be reversed in neural tube defects (NTD), where survival of male fetuses has been found to be poor compared with females, resulting in the higher risk of NTDs in females at birth (Källén et al., 1994). The interaction of sex hormones and system development has been cited as possible causes of sex differences in some anomalies including cleft palate and lip (Nagase et al., 2010). Other theories for sex differences include that the earlier time of gestation at which male reproductive organs develop and their susceptibility to excess hormone levels may account for the increased level of male urinary and reproductive defects (Lubinsky, 1997; Lary and Paulozzi, 2001), although there is little empirical evidence to support these theories.

## Conclusions

This study confirms the greater risk for males to be born with major CAs and additionally highlights substantial variation in this risk by system-specific subgroup and specific diagnosis. Sociodemographic and maternal factors that have been shown to affect the prevalence of CAs had no effect on the relationship between CAs and sex. The large population and pooled analyses in this study afforded us increased statistical confidence in many of these estimates and the nationally representative data provides generalizability to the wider population of the United Kingdom and thus may give further clues as to the causative mechanisms of CAs and means of prevention.
