# Supplementary material for: Sex prevalence of major congenital anomalies in the United Kingdom: A national population-based study and international comparison meta-analysis
Source: Birth Defects Res A Clin Mol Teratol. 2014 Feb 12;100(2):79–91. doi: 10.1002/bdra.23218 (PMC4016755; doi:10.1002/bdra.23218)
Supplement: Supplementary file 1 [file bdra0100-0079-sd1.docx]

Supplementary tables: Male to female unadjusted and adjusted odds ratios for specific congenital anomalies

|  |  |  |  |  |  |  |  |  |
| --- | --- | --- | --- | --- | --- | --- | --- | --- |
|  |  | **Unadjusted odds ratio** | | |  | **Adjusted odds ratio** | | |
|  |  | **Male : Female** | **95% CI** | |  | **Male : Female** | **95% CI** | |
| **Nervous system** |  | **1.04** | **(0.91** | **, 1.18)** |  | **1.04** | **(0.91** | **, 1.18)** |
| Hydrocephaly |  | 1.23 | (0.99 | , 1.53) |  | 1.23 | (0.99 | , 1.53) |
| Microcephaly |  | 0.95 | (0.75 | , 1.19) |  | 0.95 | (0.75 | , 1.19) |
| Neural Tube Defects |  | 0.98 | (0.67 | , 1.45) |  | 0.98 | (0.67 | , 1.45) |
| *Spina Bifida* |  | 1.07 | (0.69 | , 1.67) |  | 1.07 | (0.69 | , 1.67) |
| *Encephalocele* |  | 0.85 | (0.36 | , 2.01) |  | 0.86 | (0.37 | , 2.03) |
| Arhinen'/holoprosencephaly |  | 0.63 | (0.11 | , 3.77) |  | 0.63 | (0.11 | , 3.77) |
|  |  |  |  |  |  |  |  |  |
| **Eye** |  | **1.10** | **(0.93** | **, 1.29)** |  | **1.10** | **(0.93** | **, 1.29)** |
| Congenital cataract |  | 1.01 | (0.71 | , 1.45) |  | 1.01 | (0.71 | , 1.45) |
| Ano' / micropthalmos |  | 1.11 | (0.73 | , 1.69) |  | 1.11 | (0.73 | , 1.69) |
| *Anophthalmos* |  | 2.20 | (0.57 | , 8.51) |  | 2.21 | (0.57 | , 8.54) |
| Congenital glaucoma |  | 1.20 | (0.69 | , 2.10) |  | 1.20 | (0.69 | , 2.10) |
|  |  |  |  |  |  |  |  |  |
| **Ear, face & neck** |  | 1.12 | (0.82 | , 1.54) |  | 1.13 | (0.83 | , 1.55) |
| Anotia |  | 5.67 | (0.68 | , 47.12) |  | 5.68 | (0.68 | , 47.2) |
|  |  |  |  |  |  |  |  |  |
| **Heart** |  | **0.98** | **(0.92** | **, 1.03)** |  | **1.00** | **(0.94** | **, 1.07)** |
| Ventricular septal defect |  | 0.97 | (0.89 | , 1.05) |  | 0.97 | (0.89 | , 1.05) |
| Severe CHD |  | 1.36 | (1.18 | , 1.57) |  | 1.36 | (1.18 | , 1.57) |
| *Coarctation of aorta* |  | 1.49 | (1.13 | , 1.96) |  | 1.49 | (1.13 | , 1.96) |
| *Tetralogy of Fallot* |  | 1.05 | (0.80 | , 1.36) |  | 1.05 | (0.80 | , 1.36) |
| *Transposition of great vessels* |  | 2.12 | (1.44 | , 3.13) |  | 2.12 | (1.44 | , 3.13) |
| *Atrioventricular septal defect* |  | 0.81 | (0.51 | , 1.28) |  | 0.80 | (0.51 | , 1.27) |
| *Hypoplastic left heart* |  | 3.08 | (1.39 | , 6.80) |  | 3.08 | (1.39 | , 6.79) |
| *Pulmonary valve atresia* |  | 1.58 | (0.77 | , 3.22) |  | 1.58 | (0.77 | , 3.23) |
| *Total anom pulm venous return* |  | 1.24 | (0.60 | , 2.54) |  | 1.24 | (0.60 | , 2.55) |
| *Ebstein's anomaly* |  | 0.43 | (0.15 | , 1.24) |  | 0.43 | (0.15 | , 1.24) |
| *Common arterial truncus* |  | 2.82 | (0.29 | , 27.09) |  | 2.84 | (0.30 | , 27.3) |
| Atrial septal defect |  | 0.75 | (0.65 | , 0.87) |  | 0.75 | (0.65 | , 0.87) |
| Aortic valve atresia/stenosis |  | 2.36 | (1.40 | , 3.96) |  | 2.37 | (1.41 | , 3.97) |
| Pulmonary valve stenosis |  | 0.97 | (0.62 | , 1.54) |  | 0.97 | (0.61 | , 1.54) |
| Hypoplastic right heart |  | 1.19 | (0.32 | , 4.43) |  | 1.18 | (0.32 | , 4.40) |
|  |  |  |  |  |  |  |  |  |
| **Respiratory** |  | **1.18** | **(0.97** | **, 1.44)** |  | **1.18** | **(0.97** | **, 1.44)** |
| Choanal atresia |  | 0.79 | (0.43 | , 1.42) |  | 0.79 | (0.44 | , 1.43) |
| Cystic adenomatous malf lung |  | 1.21 | (0.32 | , 4.49) |  | 1.18 | (0.32 | , 4.40) |
|  |  |  |  |  |  |  |  |  |
| **Cleft lip or palate** |  | **1.18** | **(1.03** | **, 1.35)** |  | **1.18** | **(1.03** | **, 1.35)** |
| Cleft lip with/out palate |  | 1.61 | (1.34 | , 1.95) |  | 1.62 | (1.34 | , 1.95) |
| Cleft palate |  | 0.85 | (0.70 | , 1.03) |  | 0.85 | (0.70 | , 1.03) |
|  |  |  |  |  |  |  |  |  |
| **Digestive system** |  | **1.32** | **(1.12** | **, 1.55)** |  | **1.32** | **(1.12** | **, 1.55)** |
| Hirschsprung's disease |  | 2.51 | (1.67 | , 3.77) |  | 2.51 | (1.67 | , 3.78) |
| Ano-rectal |  | 1.77 | (1.22 | , 2.55) |  | 1.76 | (1.22 | , 2.54) |
| Oesophageal atresia |  | 1.29 | (0.82 | , 2.02) |  | 1.29 | (0.82 | , 2.02) |
| Oth. parts sm intestine |  | 0.81 | (0.43 | , 1.52) |  | 0.81 | (0.43 | , 1.52) |
| Diaphragmatic hernia |  | 1.42 | (0.51 | , 4.00) |  | 1.42 | (0.51 | , 3.99) |
| Duodenal |  | 0.41 | (0.20 | , 0.87) |  | 0.41 | (0.20 | , 0.86) |
|  |  |  |  |  |  |  |  |  |
| **Abdominal wall defects** |  | **0.85** | **(0.62** | **, 1.17)** |  | **0.85** | **(0.62** | **, 1.17)** |
| Gastroschisis |  | 0.74 | (0.51 | , 1.09) |  | 0.74 | (0.51 | , 1.09) |
| Omphalocele |  | 1.09 | (0.60 | , 1.98) |  | 1.09 | (0.60 | , 1.98) |
|  |  |  |  |  |  |  |  |  |
| **Urinary system** |  | **1.51** | **(1.36** | **, 1.68)** |  | **1.51** | **(1.36** | **, 1.67)** |
| Hydronephrosis |  | 3.30 | (2.24 | , 4.86) |  | 3.30 | (2.24 | , 4.86) |
| Renal dysplasia |  | 2.06 | (1.32 | , 3.23) |  | 2.06 | (1.32 | , 3.22) |
| Bladder exstrophy/epispadia |  | 5.07 | (2.66 | , 9.65) |  | 5.08 | (2.67 | , 9.66) |
| Posterior urethral valve |  | 16.61 | (3.99 | , 69.1) |  | 16.56 | (3.98 | , 68.8) |
|  |  |  |  |  |  |  |  |  |
| **Genital** |  | **16.8** | **(14.0** | **, 20.1)** |  | **16.8** | **(14.0** | **, 20.1)** |
| Hypospadias |  | 632 | (204 | , 1961) |  | 632 | (204 | , 1961) |
|  |  |  |  |  |  |  |  |  |
| **Limb** |  | **0.70** | **(0.65** | **, 0.75)** |  | **0.70** | **(0.65** | **, 0.75)** |
| Club foot |  | 1.22 | (1.06 | , 1.39) |  | 1.22 | (1.06 | , 1.39) |
| Hip dislocation/dysplasia |  | 0.26 | (0.23 | , 0.30) |  | 0.26 | (0.23 | , 0.30) |
| Polydactyly |  | 1.31 | (1.07 | , 1.59) |  | 1.31 | (1.07 | , 1.59) |
| Syndactyly |  | 1.73 | (1.35 | , 2.22) |  | 1.74 | (1.36 | , 2.22) |
| Other |  | 0.86 | (0.68 | , 1.08) |  | 0.86 | (0.68 | , 1.08) |
| Limb reduction |  | 0.93 | (0.71 | , 1.22) |  | 0.93 | (0.71 | , 1.22) |
| *Lower limb reduction* |  | 0.71 | (0.50 | , 1.01) |  | 0.71 | (0.50 | , 1.01) |
| *Upper limb reduction* |  | 1.44 | (0.87 | , 2.38) |  | 1.44 | (0.87 | , 2.38) |
| *Limb reduction other* |  | 1.51 | (0.69 | , 3.34) |  | 1.51 | (0.69 | , 3.34) |
| Arthrogryposis multi congenita |  | 1.41 | (0.50 | , 3.97) |  | 1.42 | (0.51 | , 3.99) |
|  |  |  |  |  |  |  |  |  |
| **Musculoskeletal** |  | **1.35** | **(1.17** | **, 1.56)** |  | **1.35** | **(1.17** | **, 1.56)** |
| Craniosynostosis |  | 4.51 | (2.82 | , 7.23) |  | 4.51 | (2.82 | , 7.21) |
| Achondroplasia |  | 1.45 | (0.81 | , 2.58) |  | 1.44 | (0.81 | , 2.58) |
|  |  |  |  |  |  |  |  |  |
| **Other** |  | **0.98** | **(0.83** | **, 1.16)** |  | **0.98** | **(0.83** | **, 1.16)** |
| Disorders of skin |  | 1.57 | (1.15 | , 2.15) |  | 1.57 | (1.15 | , 2.15) |
| Situs inversus |  | 0.60 | (0.23 | , 1.56) |  | 0.60 | (0.23 | , 1.55) |
| Asplenia |  | 1.10 | (0.37 | , 3.28) |  | 1.10 | (0.37 | , 3.28) |
| Conjoined twins |  | 0.23 | (0.01 | , 3.60) |  | 0.47 | (0.04 | , 5.22) |
|  |  |  |  |  |  |  |  |  |
| **Chromosomal** |  | **1.16** | **(1.03** | **, 1.31)** |  | **1.16** | **(1.02** | **, 1.31)** |
| Down syndrome |  | 1.05 | (0.89 | , 1.24) |  | 1.05 | (0.89 | , 1.24) |
| Klinefelter syndrome |  | - | - | - |  | - | - | - |
| Wolf-Hirschhorn synd |  | 2.50 | (0.66 | , 9.44) |  | 2.52 | (0.67 | , 9.51) |
| Turner syndrome |  | 0.09 | (0.04 | , 0.23) |  | 0.09 | (0.04 | , 0.23) |
| Edward syndrome |  | 0.32 | (0.09 | , 1.17) |  | 0.32 | (0.09 | , 1.16) |
| Patau syndrome |  | 1.24 | (0.28 | , 5.56) |  | 1.26 | (0.28 | , 5.64) |
| Cri-du-chat syndrome |  | 0.59 | (0.10 | , 3.55) |  | 0.63 | (0.11 | , 3.77) |
|  |  |  |  |  |  |  |  |  |
| **Genetic** |  | **1.10** | **(0.91** | **, 1.32)** |  | **1.10** | **(0.91** | **, 1.32)** |
|  |  |  |  |  |  |  |  |  |
